# The role of amino acid metabolism in inflammatory bowel disease and other inflammatory diseases

**DOI:** 10.3389/fimmu.2023.1284133

**Published:** 2023-10-23

**Authors:** Xiaowen Zheng, Yi Zhu, Zihan Zhao, Ying Chu, Wenjing Yang

**Affiliations:** ^1^ Key Laboratory of Medical Science and Laboratory Medicine of Jiangsu Province, School of Medicine, Jiangsu University, Zhenjiang, China; ^2^ The People’s Hospital of Danyang, Affiliated Danyang Hospital of Nantong University, Zhenjiang, Jiangsu, China; ^3^ Changzhou Key Laboratory of Molecular Diagnostics and Precision Cancer Medicine, Wujin Hospital Affiliated with Jiangsu University, Changzhou, Jiangsu, China

**Keywords:** inflammation, inflammatory bowel disease, amino acid metabolism, mesenchymal stem cell, exosome

## Abstract

Inflammation is a characteristic symptom of the occurrence and development of many diseases, which is mainly characterized by the infiltration of inflammatory cells such as macrophages and granulocytes, and the increased release of proinflammatory factors. Subsequently, macrophage differentiates and T cells and other regulated factors exhibit anti-inflammatory function, releasing pro- and anti-inflammatory factors to maintain homeostasis. Although reports define various degrees of metabolic disorders in both the inflamed and non-inflamed parts of inflammatory diseases, little is known about the changes in amino acid metabolism in such conditions. This review aims to summarize amino acid changes and mechanisms involved in the progression of inflammatory bowel disease (IBD) and other inflammatory diseases. Since mesenchymal stem cells (MSCs) and their derived exosomes (MSC-EXO) have been found to show promising effects in the treatment of IBD and other inflammatory diseases,their potential in the modulation of amino acid metabolism in the treatment of inflammation is also discussed.

## 1 Introduction

Inflammation is the activation of immune cells and other non-immune cells during the defense against harmful invasion of life, protecting the host from bacteria, viruses, toxins, and infections by eliminating pathogens and promoting tissue repair and recovery. The normal inflammatory response is timely. However, inflammation for a long time can lead to chronic inflammation and a range of autoimmune diseases. The occurrence and development of disease are mostly accompanied by inflammation whose main feature is significantly increased expression of pro-inflammatory cytokines such as interleukin (IL) - 1β, IL - 6, TNF -α, etc. (1), leading to the anti-inflammatory and pro-inflammatory cytokines imbalance, promoting the incidence of inflammatory and autoimmune diseases including IBD and rheumatoid arthritis (RA) (2). How to eliminate inflammation in the later stage of the disease is the focus of experimental research and clinical treatment. Inflammatory bowel disease (IBD) is a non-infectious, chronic, systemic, and recurrent inflammatory disease of the gastrointestinal tract. IBD is characterized by recurrent and prolonged episodes of diarrhea and abdominal pain, mainly including ulcerative colitis (UC) and Crohn’s disease (CD). From the aspects of immunity, IBD can be regarded as a kind of inappropriate immune response. IBD is characterized by recurrent and prolonged episodes of diarrhea and abdominal pain and can affect any location throughout the gastrointestinal tract. IBD not only affects the quality of life, work, and leisure but also greatly increases the risk of colorectal cancer (3). This disease is not only widespread in the West but also a problem for many people in Asian countries, including China. At present, there is no specific therapy or drug to completely cure IBD, and most treatments only target its clinical symptoms to relieve it. The recurrence of the disease brings economic and mental burdens to the patients and their families, so it is urgent to actively seek effective treatment. The pathogenesis of IBD is still unclear and involves complex factors such as genetics, environment, and immunity. More studies have reported that IBD is associated with intestinal mucosal integrity, permeability and barrier function, intestinal immune status, intestinal flora, gastrointestinal anti-inflammatory cytokine and pro-inflammatory cytokine balance, and intestinal oxidative stress.

Amino acids are an important class of substances, mainly composed of five elements, which are carbon, hydrogen, oxygen, nitrogen, and sulfur. There are 20 amino acids known to make up proteins in the human body. The biological functions of amino acids include basic units of protein, energy metabolizers, precursors of nitrogen compounds, etc. The essential amino acid/non-essential amino acid ratio in the blood is usually 3-3.5. Studies have shown that this ratio can change in disease, and some amino acid levels can also be affected (4, 5). Amino acid metabolism is an important process of normal life activities, including deamination, decarboxylation, transamination, ammonia metabolism, oxidation energy supply, and other general amino acid metabolism processes as well as individual amino acid metabolism, taking branched-chain amino acid metabolism as an example (4, 6). Exogenous amino acids (produced by digestion and degradation of protein in food) and endogenous amino acids (produced by the degradation of tissue protein in the body) are distributed throughout the body, known as the amino acid metabolic pool. As an important component of metabolism, amino acids are not only used for protein synthesis but also participate in important human life activities mainly through amino acid catabolism. For example, some amino acids can be decarboxylated under the action of a specific amino acid decarboxylase to produce corresponding amines. The amines produced after amino acid decarboxylation are low in content but have important physiological functions. For example, glutamic acid (Glu) decarboxylates and then generates gamma-aminobutyric acid (GABA), an inhibitory neurotransmitter which has a suppressive effect on the central nervous system (7); Histamine produced by histidine (His) catalyzed by histidine decarboxylase is widely distributed in the body, with strong vasodilation function, and histamine is released at the wound site and inflammation site (8). Another example is tryptophan (Trp) catalyzed by tryptophan hydroxylase (TPH) to produce 5-hydroxytryptamine (5-HT), which is widely present in the gastrointestinal tract and nervous tissue (9). 5-HT acts as a neurotransmitter in the brain and has the function of constricting blood vessels in peripheral tissues. It can be seen that amino acids and their metabolites are important participants in human life activities.

Exosomes are nano-scale vesicles that can be secreted by a variety of cells. They are about 30 to 150nm in diameter, and their composition varies according to their origin. Exosomes are spherical bilayer lipid particles containing various types of proteins, lipids, DNA, non-coding RNA, miRNA, and mRNA, which can cause the exchange and reprogramming of genetic information in recipient cells, that is, have regulatory effects on target cells (10). The typical representatives of exosome proteins are annexin and G protein, which is closely related to trans-membrane transport and is also the driving factor that determines the secretion of exosomes into cells through membrane fusion. Exosomes express biomarkers such as CD63, CD9, and CD81 on the surface (11). Since exosomes show therapeutic effects as transfer vectors and participate in immune system regulation, more and more studies have been conducted to explore the relationship between exosomes and diseases. For example, In one study, colonic epithelial-derived exosomes carrying transforming growth factor-β1 (TGF-β1) exhibited immuno-suppressive activity by inducing both Treg and immuno-suppressive dendritic cells (DC) to treat colitis (12). Exosomes also serve as good drug carriers to deliver proteins, mRNA, and other biological macro-molecules or active substances. Thus, exosomes are active natural nanocarriers that can be used to provide innate anti-inflammatory biological components for the treatment of IBD (13).

This review mainly summarizes the changes in amino acid metabolism in the pathogenesis of IBD and other inflammatory diseases. And the review additionally talks about what effects MSCs and their derived exosomes have on the treatment in inflammatory diseases and aims at finding some novel therapeutic targets of this method.

## 2 Amino acids and immune cells

Some amino acid catabolic pathways have become key checkpoints of immunity (14, 15).

### 2.1 Amino acids and the regulation of macrophages

**Figure 1:**
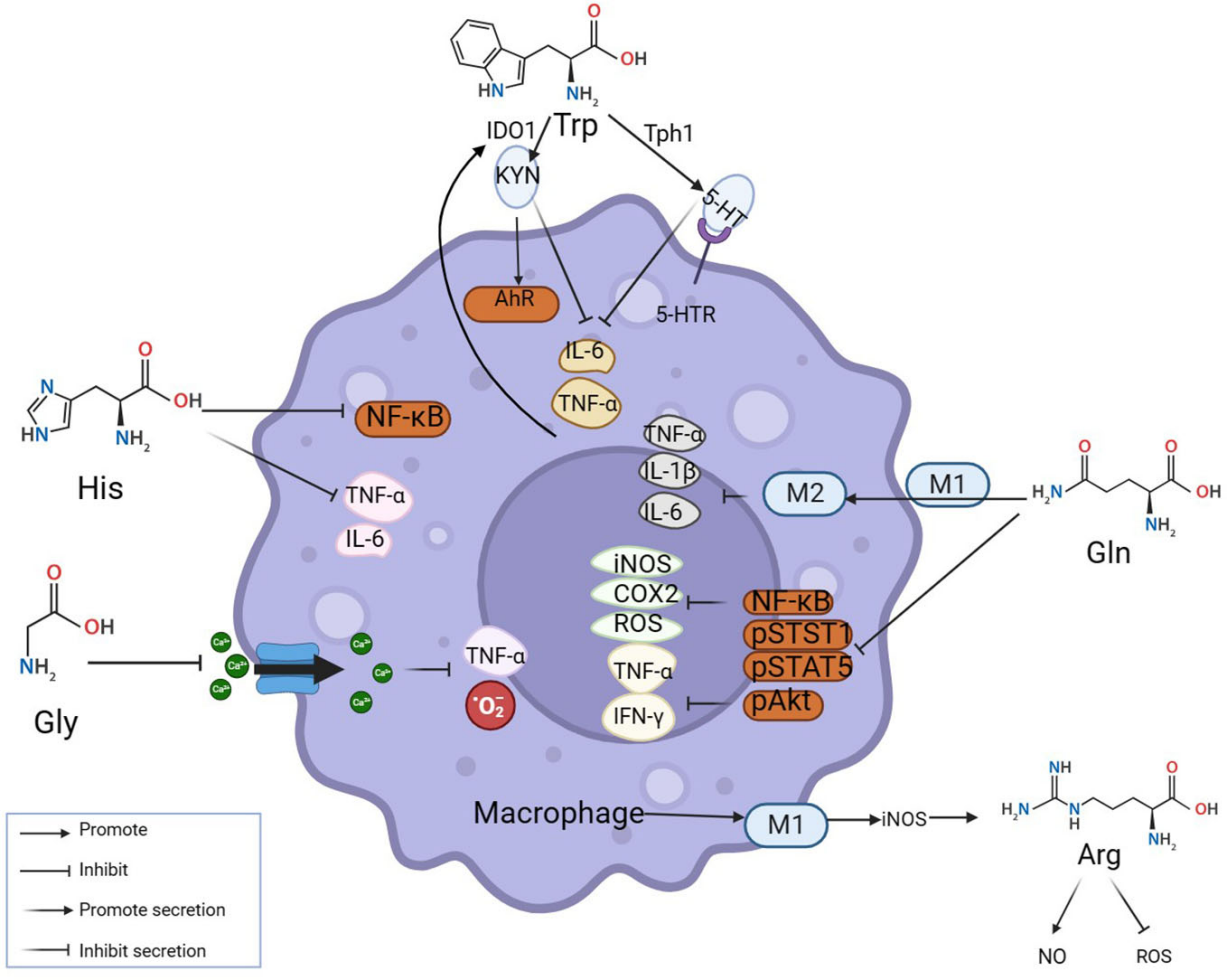
Amino acids modulate inflammation by reciprocal regulation of macrophages. The mechanisms at inflammatory sites by which amino acids exert anti-inflammatory effects by interacting with macrophages are illustrated above. Amino acids make an impact on macrophages by mediating their polarization and secretion. Many classic inflammatory pathways in macrophages, such as NF-κB, STAT1, and STAT5, are inhibited by amino acids.

Macrophages are key cells in the occurrence and development of inflammatory diseases (16). They maintain the internal environment by polarization into pro-inflammatory macrophages M1 and anti-inflammatory macrophages M2 and regulating the balance of pro-inflammatory/anti-inflammatory cytokines. At the site of inflammation, amino acids as vital substances contribute greatly to the nodes involved in the elimination of inflammation. Relevant studies and literature over the years indicate that the participation of amino acids in eliminating the inflammatory process can be achieved through mutual regulation with macrophages (**Figure 1**). Several major amino acids and their metabolites directly or indirectly inhibit the levels of inflammatory factors such as TNF- α, IFN- γ, and IL-6 by promoting M2 polarization, inhibiting NF- κ B, STST 1, and STAT 5, and simultaneously reducing the levels of ROS, iNOS, and COX 2 in macrophages (**Figure 1**). As shown in the figure, Trp acts on 5-hydroxytryptamine receptor (5-HTR) expressed in macrophages through catabolism leading to serotonin (5-HT), inhibiting the release of pro-inflammatory factors by macrophages (17, 18); Under inflammatory conditions, pro-inflammatory factors such as TNF-α can up-regulate indoleamine 2, 3-dioxygenase 1(IDO1) and then increase the activity of Trp catabolism leading to kynurenine (KYN) (19). Histidine inhibits NF-κB activity in a concentration-dependent manner and then reduces the release levels of TNF- α, and IL-6 in macrophages (20). Glutamine inhibits the production of ROS, NOS, iNOS, COX-2, and other pro-inflammatory mediators to protect tissue (21). Reducing the level of NF-κB activation in inflammation while simultaneously inhibiting STAT1, STAT5, and Akt phosphorylation, inhibiting the increase of TNF-α and IFN-γ (22). Glu also promotes the conversion of M1 to M2 in muscle, down-regulating IL-1β et al. (23). Glycine (Gly) blunted calcium influx in macrophages to inhibit the production of toxic free radicals by macrophages (24). Additionally, Macrophage M1 polarization promotes iNOS expression and stimulates arginine to generate NO while inhibiting ROS activity (25). Thus, amino acids can function by interacting with macrophages.

### 2.2 Amino acids and the regulation of T cells

T lymphocytes are also important players and balancers at the sites of inflammation. Among them, CD8 + T cells receive antigen signals submitted by major histocompatibility complex I (MHCI) to directly eliminate pathogens or tumor cells through cytotoxic effects. Therefore, it is considered the key to the anti-tumor immune response (26). CD4 + T cells mainly receive the presenting signal of major histocompatibility complexII (MHCII) to eliminate the antigen by releasing various cytokines. There are many subsets of CD4 + T cells, among which the Th subset is important in the pathogenic process of inflammatory diseases, capable of mediating a variety of chronic inflammation and autoimmune diseases. For example, the Th 17, which has been discovered for more than a decade, is indispensable for the onset of IBD and is also found to be actively involved in experimental autoimmune encephalomyelitis (EAE) by releasing IL-17 (27). However, CD4+ T cells can differentiate into regulatory T cells (Tregs). As immunosuppressive T cells, Tregs can inhibit the activation of other T cells and become a mainstay in controlling the proper immunity of the body under normal physiological conditions (28). Tregs have been proven to be dysfunctional when the body experiences chronic inflammation or autoimmune diseases, so Tregs have become an important regulatory target to deal with excessive immune responses (29, 30). In recent years, studies on treating inflammatory diseases by regulating Th 17/Treg levels have emerged. Researchers use many methods to restore the balance of the Th 17/Treg axis to regulate the inflammatory response (31–33).

**Figure 2:**
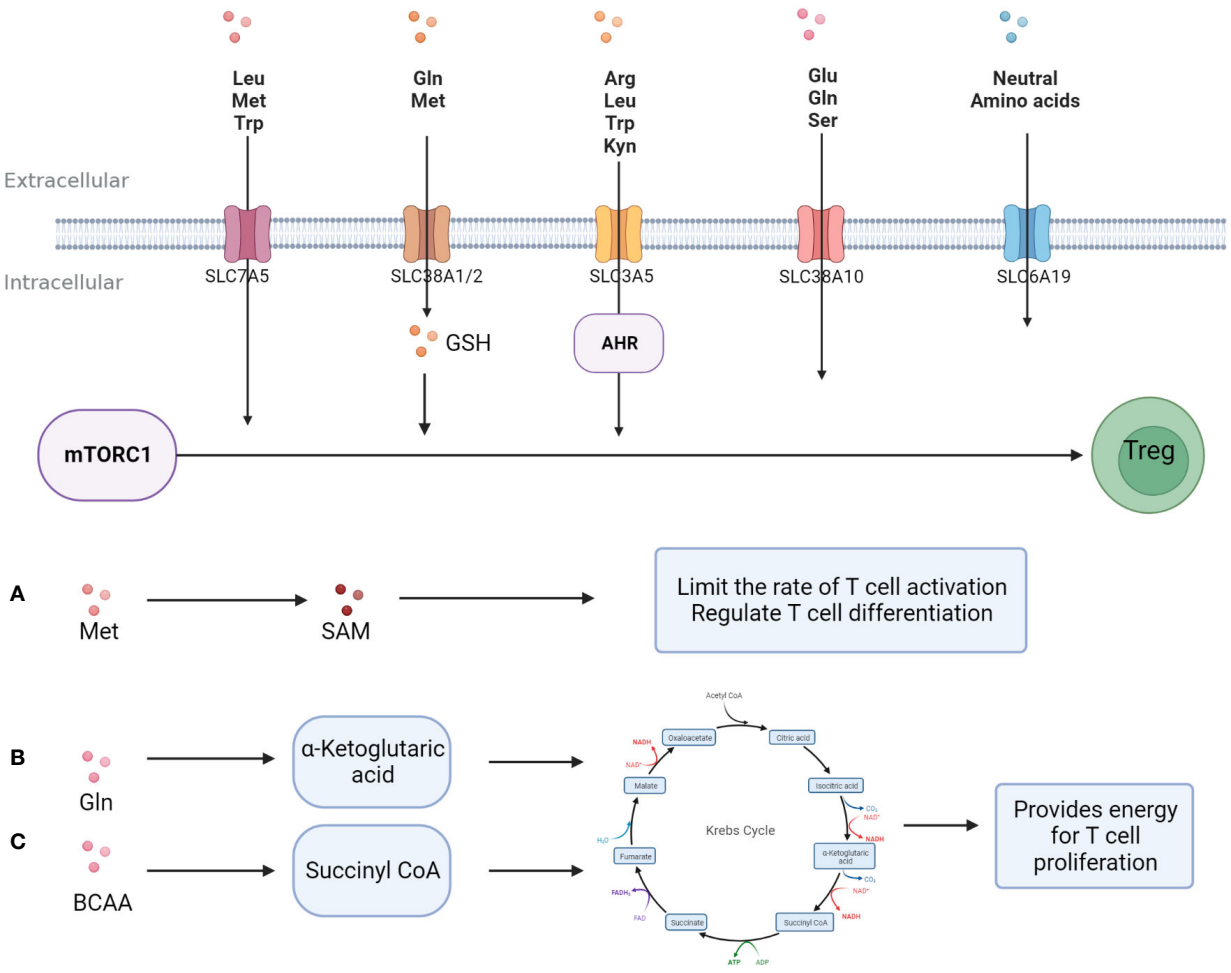
Amino acids promote T -cell proliferation and differentiation. Amino acids enter T cells and support T cell proliferation and differentiation in three major ways; **(A)** A variety of amino acids activates mTORC1 directly or indirectly after entering T cells, promoting Treg differentiation and activity. **(B)** Methionine is metabolized to S-adenosine methionine, provides a substrate for methylation, and regulates the typing and activity of T cells. **(C)** Some amino acid metabolites are involved in glucose metabolism and provide energy for T cell proliferation.

Amino acids and their metabolites play an important role in the growth and function of T cells. First, amino acids and their metabolites can participate in T cell internal nutrient metabolism through the intracellular entry of various amino acid transport proteins widely expressed on the T cell membrane to maintain T cell survival and proliferation. For example, glutamine (Gln) enters T cells through Recombinant Solute Carrier Family 1, Member 5 (SLC1A5)/Recombinant Solute Carrier Family 38, Member 1(SLC38A1) and is metabolized to α-ketoglutarate, and then participates in the intra-cellular TCA cycle to generate ATP to provide energy for T cell proliferation (34). Arginine (Arg) enters the cell through SLC7A1 and can generate polyamines to maintain the needs of T cell growth (35). Secondly, the mammalian target of rapamycin (mTOR) and general control nonderepressible 2 (GCN2) are important amino acid sensors. Various amino acids and their metabolites, such as leucine (Leu), arginine (Arg), and glutathione (GSH), regulate the proliferation and differentiation of T cells by activating the amino acid sensor mTORC1 (36). Trp and its metabolite kynurenine (Kyn) can enter T cells through Solute Carrier Family7, Member5 (SLC7A5) and activate intra-cellular GCN2 to inhibit Th 17 differentiation (37), and T cell proliferation is partly dependent on the activity of GCN2 (38). In addition, amino acid post-translational modifications, such as methylation, acetylation, etc., are important players in directly regulating T cell typing from the genetic level. The methionine (Met) metabolite S-adenosine methionine (SAM) is the main methyl donor (39), while Gln and branched-chain amino acids can be metabolically metabolized to CoA to provide acetyl groups (40) (**Figure 2**).

## 3 Amino acid metabolism and the progression and treatment of inflammatory diseases

### 3.1 Amino acids and the pathogenesis of IBD

The body’s metabolism is disturbed by a pathological state, and amino acid metabolism is no exception. In recent years, there has been constant new evidence that amino acid metabolism is closely related to inflammation (**Table 1**). A randomized controlled trial of the relationship between branched-chain amino acids and inflammation directly revealed this (52). A large number of studies have shown that compared with the normal state, in the course of inflammatory diseases, the serum levels of 20 amino acids are reduced to varying degrees (5, 53), with glutamic acid, arginine, glycine, etc. being the most significant. Metagenomic analysis showed major perturbations in amino acid metabolism in IBD: the abundance of metabolic and biosynthetic genes decreased for almost all amino acids (especially histidine and lysine), while the abundance of arginine, histidine, and lysine transporter genes increased (54). These findings indicate that amino acid metabolism is affected by IBD pathological status. **Table 2** summarizes some amino acid changes during the IBD process (**Table 2**). Mingyan Jing et al. reviewed the real location of amino acids in the body under inflammation or disease, especially mentioning that threonine is closely related to intestinal metabolism and plays an important role in maintaining the non-specific immune barrier of intestinal mucosa (6).

**Table 1:** Relationship between amino acid metabolism and inflammatory diseases.

Disease	Key findings	Significance	Reference
Coronary artery disease CVD (e.g., Atherosclerosis)	L-Arg is positively correlated with inflammatory markers in CVD, but L-Arg is the only precursor of NO able to alleviate the inflammatory process in CVD. L-Arg supplementation may be beneficial for early atherosclerosis. However, in advanced atherosclerotic conditions, arginase expression increases, nitric oxide synthase expression decreases, and L-Arg-NO pathway balance is disrupted	Treatment	(41–43)
Multiple sclerosis (MS)	The amino acid catabolism of Trp and Arg is impaired in MS patients	Mechanism	(44)
Rheumatoid Arthritis (RA)	Peripheral CD14 mononuclear macrophages in RA patients were activated and increased SLC7A5 expression, which mediated leucine and other influx, enhanced mTOR activity, and increased the levels of IL-1 β and TNF-α in macrophages	Mechanism	(45)
Inflammatory bowel disease (IBD)	Glutamine treatment was shown experimentally to repair epithelial tissue and restore cell permeability, but no improvement in clinical symptoms was seen	Therapy	(22, 46, 47)
	Histidine in the IBD model was able to inhibit NF-κB activation and reduce TNF- α as well as IL-6 and other proinflammatory factors in a concentration-dependent manner	Therapy	(20)
Systemic lupus erythematosus (SLE)	GSH depletion in SLE patients accumulated canine urine in lymphocytes and stimulated mTORC1, resulting in the expansion of DNT cells and elevated expression of IL-4 and IL-17	Mechanism	(44, 48, 49)
Non-alcoholic fatty liver disease (NAFLD)	Circulating valine excess disrupts amino acid metabolism and fatty acid metabolism thereby promoting NAFLD by inhibiting mTORC2	Mechanism	(50)
	Serine supplementation decreased hepatic inflammation response, oxidation products and protected GSH antioxidant systems in HFD-induced mice	Therapy	(51)

**Table 2:** Perturbation of amino acid metabolism in IBD.

Amino acid species	Related enzymes/ transporter proteins	Relevant metabolism pathways	Changes in IBD	Reference
Trp	IDO1/SLC6A19	Trp-Kyn/Trp-5-HT	Both the serum and fecal Trp levels were decreased;	(55, 56)
			Intestinal epithelial SLC6A19 deficiency causes a significant reduction in tryptophan uptake;	(57)
			A decrease in the tryptophan metabolite receptor AHR activation	(58)
Gln	SLC38A2	Gln-mTOR (+)/NF-κB(-)	The Gln levels were significantly decreased in both UC and CD patients	(55, 56)
Phe	phenylalanine-4-hydroxylase (a hepatic enzyme)		The concentrations rise in the serum	(59, 60)
Arg	CAT-2(SLC7A2)		The concentrations rise in the serum	
Gly	GLYT1		Gly concentration increased in the urine ;	(59)
			Supplement with exogenous glycine can prevent IBD	(60)
Glu	SLC1A2	Glu-MAPK	DSS stimulated elevated serum Glu concentrations in rats	(59)
Ile	SLC16A14	L-Ile-TLR4 / MyD88 / NF-κB (-)	Administration treatment for IBD	(61)
His	SLC7A1/SLC7A7		Histidine phosphorylation increases in colitis	(62)

The symbol "+" means positive regulation and "-" means negative regulation.

A typical example is 5-HT. 5-HT is an important regulator in the human body, derived from the catabolism of tryptophan. More than 90% of 5-HT in the human body is produced in the gut, specifically in intestinal chromaffin cells (ECs), a specific subtype of intestinal epithelial cells (63). This process occurs through the action of the Trp hydroxylase 1 enzyme (TpH1), which produces 5-hydroxytryptophan (5-HTP), and is further metabolized into 5-HT (64). Under physiological conditions, peripheral 5-HT does not cross the blood-brain barrier. Peripheral 5-HT triggers many functions in the gastrointestinal tract and is associated with a wide range of human physiological functions through the activation of specific 5-HT receptors. Specifically, 5-HT is an important gastrointestinal signaling molecule that transmits signals from the gut to internal or external neurons and influences intestinal peristalsis and movement, secretion, vasodilation, and nutrient absorption (58).The intestinal microbiota is a major player in intestinal 5-HT production (65). Its effect has been demonstrated in germ-free mice that exhibit impaired 5-HT production in the colon (but not in the small intestine) and low 5-HT concentration in the blood. The mechanism by which intestinal microbiota regulates 5-HT production 3 is not fully understood, but the role of SCFAs in stimulating TpH1 expression has been proposed (66). In addition, some secondary bile acids, such as deoxycholate produced by microbial bioconversion of cholate, can also stimulate 5-HT biosynthesis. Most studies (67–70) report a decrease in intestinal microbiome diversity in patients with IBD, and this change is considered to be an important factor in the development of IBD (54, 71, 72). IBD patients have obvious intestinal flora imbalance, which is mainly manifested as a decreased abundance of intestinal flora, decreased stability of intestinal dominant flora, decreased protective flora, and increased pathogenic bacteria. Intestinal inflammation is usually associated with a significant decrease in Bacteroides (gram-negative microorganisms) and Firmicutes (gram-positive) populations (67, 68), and an increase in the ratio of gram-negative to gram-positive microbes in the gut leads to an increase in the release of inflammatory LPS (73).

### 3.2 Amino acids and the treatment of IBD

It has been reported that glutamine can reduce the damage caused by oxidative stress in DSS-induced colitis mice (74). Mechanistically, glutamine can up-regulate the expression of tight junction proteins and maintain intestinal barrier integrity (75); Meanwhile, glutamine can also inhibit the activity of NF-κB and STAT and increase the stability of IκB, and this may be related to the increased expression of HSP 70 and HSP 25 (76). There is also a conjecture that glutamine may influence the production of IL-8, thereby targeting intestinal inflammation (77). Arginine can stimulate the synthesis of collagen, reduce the expression of iNOS, and reduce the level of pro-inflammatory cytokine IL-17 (74) so that the improvement of the colitis mouse model can be observed. There have been several articles on dietary treatments for IBD that have demonstrated the involvement of exogenous amino acids in improving the intestinal environment. Amino acid-balanced diet-fed mice can effectively alleviate inflammation and this effect is associated with amino acid interaction with gut microbes (78). Thus, amino acids and their metabolites have strong links with intestinal health.

Some special amino acids play a key role in inflammation and immune response (6). Nikolaus et al. found that changes in Trp metabolism are closely related to the progression of IBD (79). Allison et al. also found that Trp metabolism is associated with host intestinal inflammation, epithelial barrier, and energy homeostasis in IBD (80), and is closely related to the intestinal flora. Intestinal microbes such as Bacteroides and *Fusobacterium* have been shown to act as modulators of intestinal tryptophan metabolism (81). Examples include *Clostridium*, which is involved in Trp decarboxylation and leads to the production of the neurotransmitter tryptamine (64). *Escherichia coli* and *Lactobacillus*, which directly convert Trp to indole and its derivatives, are key to enhancing intestinal AHR activity. AHR signaling is considered to be a key component of the barrier site immune response and is essential for intestinal homeostasis (82, 83).. The AHR also plays an extremely important role in controlling the inflammatory function of macrophages. AHR can be expressed in microglia. A study showed that loss of microglial AHR aggravates inflammation in the central nerve system in mice (84) and dietary amino acid deficiency further aggravates the symptoms of autoimmune encephalomyelitis. Previous studies have also demonstrated that damage to AHR ligand, equally tryptophan metabolite, is detected in patients with IBD (82, 85), resulting in decreased activation of AHR in intestinal epithelial cells and immune cells, thus affecting intestinal homeostasis. Studies have shown that CARD 9, a susceptibility gene for IBD, promotes the recovery of colitis by promoting the production of interleukin (IL) -22, and that CARD 9-/-mice are more likely to develop colitis. The microbiota from Card9-/-mice cannot metabolize tryptophan as a metabolite acting as a ligand for the aryl hydrocarbon receptor (AHR) (85). This may directly indicate that the Trp-AHR pathway is a key therapeutic target in colitis. In addition, other tryptophan metabolites also play an important role in the process of anti-inflammatory. For example, under the catalysis of tryptophan decarboxylase (TrpD), tryptophan oxygenase - 2 - sheet (TMO), and bacteria tryptophan gradually turns into indole - 3 - acetic acid (IAA) and indole - 3 - propionic acid (IPA) which even have direct anti-inflammatory effects (86).

It has been found that in the large intestine, *Escherichia coli* participates in the process of amino acid fermentation, mainly using lysine, arginine, glycine, and branched amino acids as substrates to produce a large number of complex metabolites including ammonia, acetic acid, butyric acid, and branched fatty acids. These products have been shown to affect the physiological function of intestinal epithelial cells by affecting their signaling pathways and regulating the mucosal immune system. Butyric acid (87), in particular, plays such major roles and is a major source of energy for intestinal lining cells. Butyric acid provides 90 percent of its total energy requirements. These cells need this SCFA to perform their important functions, especially maintaining the integrity of the intestinal wall (88). The intestinal wall is very important because it acts as a barrier between the intestinal environment and the rest of the body.

All the above examples illustrate that intestinal flora plays an important role in the metabolism of amino acids in the process of IBD. Intestinal bacteria can decompose proteins into their constituent amino acids, which are then absorbed by the human body and used in various metabolic processes. Under the condition of inflammation, intestinal flora may affect the bioavailability of amino acids (67). Therefore, it is necessary to attach importance to the regulation of the intestinal microbial environment in future research concerning the treatment of IBD.

Several studies (89, 90) report that there is a link between amino acid metabolisms and IBD process, which may be realized through various signaling pathways. There are many examples, such as glutamine and glutamate, which promote the proliferation of intestinal epithelial cells by regulating the activity of the mTOR pathway, activate the MAPK pathway to promote the proliferation and differentiation of intestinal epithelial cells, increase the expression of tight junction protein, and maintain the stability of intestinal mucosa to ensure its barrier function. Glutamine can regulate NF-κB signal transduction and STAT signaling pathway activation, to inhibit the release of IFN-γ, TNF-α, and other pro-inflammatory cytokines and reduce intestinal inflammatory response. Hsp70 has been reported to inhibit the production of LPs-stimulated inflammatory mediators such as TNF-α, IL-1, and inducible nitric oxide synthase (iNOS) (91). However, another study on the signal transduction pathway of extracellular heat shock protein HSP70 found that an exogenous (e.g., Exosome-derived) HSP70-induced signaling cascade uses TLR2 and TLR4 to activate NF-κB activity (92). However, no adverse effects caused by HSP70 have been found in the treatment of IBD. Although there are no reports, we may assume that exosomes HSP70 can be antagonized by amino acids such as glutamine in the gut to avoid the activation of NF-κB and the up-regulation of the release of TNF-α, IL-1β, IL-6, and other pro-inflammatory factors.

Amino acid transporters are essential in amino acid absorption in the gut. As early as 2006, some scholars discovered that the L-Arg transporter CAT-2 (SLC7A2) could regulate macrophage function by controlling the activities of NOS2 and arginase, thus regulating gastrointestinal inflammation (93). Recovery of the inflammatory-injured colonic epithelium also depends on the ability of CAT2 to transport L-Arg into the cells (94). Previously reported mutations in the neutral amino acid transporter B^0^AT1(SLC6A19) in Hartnup disease may result in an almost complete lack of tryptophan absorption in the gut (95). In addition to being regulated by the B^0^AT1 transporter, tryptophan absorption is also mainly regulated by the neutral amino acid transporter B^0,+^AT(SLC6A14) and the aromatic amino acid transporter TAT1(SLC16A10) (96), but the extent to which this is impaired remains to be determined. It has been reported that mRNA of neutral amino acid transporter SLC6A19/B^0^AT1 is significantly down-regulated in biopsies of patients with active CD and UC (79). It is easy to speculate that when inflammation occurs, the intestinal mucosa is damaged, permeability is damaged, and amino acid transporter expression is decreased, in particular the tryptophan transporters, which further increases the degree of intestinal inflammation. Tryptophan is also an upstream regulator of the recovery process of intestinal inflammation (97, 98). Voluntary ingestion of exogenous tryptophan can control the expression of antimicrobial peptides in the mouse small intestine and affect the regeneration and repair of the damaged intestinal epithelium.

There are many key enzymes and rate-limiting enzymes in amino acid metabolism, which may also be therapeutic targets for diseases. For instance, biopsies of the diseased colon in patients with IBD significantly express IDO1mRNA (99). Pro-inflammatory cytokines, including IFN-γ, TNF-α, IL1β, IL2, IL6, IL18, and IFN-α induce IDO1 expression in chronic inflammatory conditions. IDO1 plays an important role in immune regulation under pathophysiological conditions, and is one of the most overexpressed genes in CD, mediating potent anti-inflammatory effects through tryptophan metabolism along the Canine uric acid pathway (100).

Amino acid metabolism demonstrates an integral role in disease progression, and the importance of the gut microbiome in gut metabolism cannot be overemphasized. There are still some mechanisms that have not been clarified in the progression and treatment of IBD. For example, the microbial community involved in intestinal amino acid metabolism is regulated by the IBD susceptibility gene NOD2, which may indicate that intestinal amino acid metabolism is more regulated at the genetic level. However, due to fewer studies, this remain s to be fully understood.

### 3.3 Amino acids and other inflammatory diseases

An article on patients with rheumatoid arthritis, spinal joint disease, and osteoarthritis reported that glycine in dog urine (endogenous metabolites of tryptophan) in inflammatory state local defects (101). Sun etc. summarized the immunomodulatory effects of several specific amino acids, such as glutamine and glycine (102). These examples indicate that amino acids have great potential in the treatment of these inflammatory diseases.

Rheumatoid arthritis characterized by synovial inflammation is a kind of autoimmune disease. Although the etiology of RA is not fully evident, it is clear that amino acid metabolism disorders definitely engage in the chronic inflammation of later RA (103). Several amino acids and amines including citrulline, succinate, and glutamine were reported to be potential biomarkers for RA (103). According to a study, compared with healthy people, RA patients’ serum analysis shows the kynurenine pathway is active (104). Trp content decreased at the same time the dog urine glycine content increased significantly. One of the most obvious factors is the IDO1 increased significantly in RA (105). IDO1 plays a protective role in RA. Research has shown that IDO1 deficiency can exacerbate tissue damage (106), and the kynurenine pathway mediated by IDO1 can inhibit T cell immune response (107). However, the process of IDO1 in RA is not static from beginning to end. In the inflammatory synovial region, hypoxia conditions actually lead to a decrease in IDO1 levels (108), while IFN-γ expressed by extensive immune cells in the inflammatory environment can induce IDO activation. Therefore, the detection of IDO1 activation in RA patients may be a forward regulation of inflammation development.

Multiple sclerosis(MS) is a chronic inflammatory disease of the central nervous system. Similar to RA, the two most representative regulatory amino acids are Trp and Arg (109). Amino acids have long been found to be necessary in the activation of the mTOR pathway (110). In multiple sclerosis (MS), IDO1 and ARG1 defects cause a decline in the catabolism of Trp and Arg, increase mTOR activity, and decrease Treg and proinflammatory factor increases (44). Although amino acids have been recognized as key inputs in this pathway, the mechanism of action of the mTORC1 amino acid sensor is still not clear (111).

Systemic lupus erythematosus (SLE) is a chronic autoimmune disease characterized by abnormal responses to self-antigens (112). In the SLE process, the most significant immunol regulatory amino acid metabolism is Glutamine catabolism. Glu catabolism is essential to T cell immune response in SLE (113). A research pointed out that glutaminolysis enzymes are significantly elevated in CD4+T cells from lupus-prone TC mice (114). Therefore, inhibiting overwhelming glutaminolysis may be an important target for treating SLE (115).

## 4 Exosomes modulate inflammation partly through the regulation of amino acid metabolism

Currently, antibiotics, anti-inflammatory drugs (e.g., 5-aminosalicylic acid, corticosteroids), or immunosuppressants (e.g., azathioprine, 6-mercaptopurine) are used to treat IBD (116). Some of these drugs are effective at relieving early inflammatory symptoms, but their long-term effectiveness is compromised by toxic buildup. Nanoparticle-based (NP) drugs are widely studied for their potential to solve such problems (13). The exosomes derived from mesenchymal stem cells (MSC-EXO) have attracted much attention. Exosomes have been widely studied for the treatment of clinical diseases due to their low immunogenicity and biocompatibility. In recent years, people have begun to pay attention to the physiological and pathological functions of exosomes and their complex components. Recent studies have shown that exosome cross-talk mechanisms may influence major IBD-related pathways such as immune response, barrier function, and intestinal flora (117, 118). For example, previous studies have shown that canine adipose tissue-derived MSC extracellular vesicles increase the polarization of M2 macrophages and Tregs *in vitro* through TSG-6 (119–121). MSC- EXO has been shown to play immunosuppressive and intestinal barrier repair functions in IBD by secreting the protein TSG-6 (122, 123). Moreover, exosomes regulate macrophage differentiation to modulate the level of cytokines and protein expression (124). IL-7 is considered to be an important cytokine in the activation of mucosal inflammation in the progression of IBD and Tao Ji et al. found that in DSS-induced colitis mice, the expression of AHR is down-regulated, while IL-7 from epithelium is up-regulated, indicating that the AHR-driven signal may regulate IL-7 (125). In experimental colitis, the AHR agonist beta-naphthalene flavone attenuates the symptom of colitis and reduces the response of human epithelial colon cells induced by LPS treatment, suggesting that AHR activation in epithelial cells may be an important mechanism regulating intestinal inflammation (126–128). Mao et al. found that exosomes derived from human umbilical cord MSCs (hucMCSs) may restore enterocyte proliferation capacity (129) by reducing macrophage infiltration in colon tissue and down-regulating NOS as well as IL-7 expression in macrophages. It is therefore reasonable to speculate that human umbilical cord mesenchymal stem cell-derived exosomes can also act on aromatics receptors, probably through the AHR ligand tryptophan. Moreover, the ultraviolet irradiation product of tryptophan is also one of the ligands with a high affinity to AHR (128).

Aside from some of the assumptions about the effect of MSC-EXO in IBD, there is real evidence holding that in other diseases. A recent research shows that hucMSC-EXO can increase the level of GSH to improve non-alcoholic steatohepatitis (NASH) although the increase is not direct (130). Another research reveals that Bone marrow MSC-derived exosomes (BMSC-EXO) regulate the level of chondrocyte glutamine metabolism and then alleviate Osteoarthritis (OA) (131).

**Figure 3:**
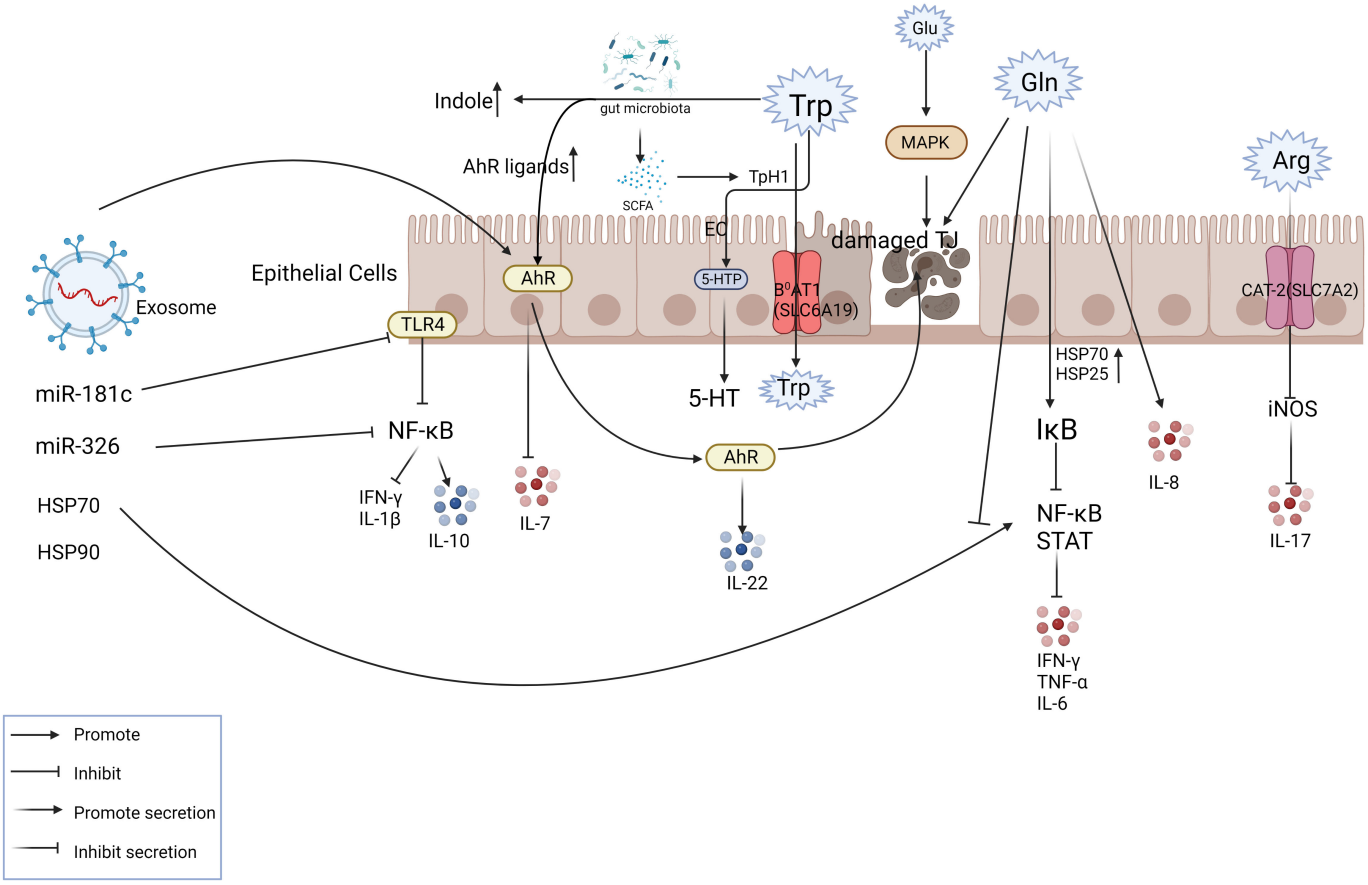
Exosomes play a role in mitigating IBD partly via amino acid metabolism. The figure shows the transport mode of amino acids inside and outside the intestinal epithelial cells with some of the key nodes involved in IBD. The involvement of exosome components in intestinal amino acid metabolism plays a therapeutic role at the site of inflammation. For example, exosomes can activate AHR, inhibit IL-7 levels, promote IL-22 release, and relieve inflammation.

Non-coding RNA in exosomes plays an important role in anti-inflammatory. One study showed that MSC-derived exosome miR-326 reduced the neddylation of IκB, inhibiting NF-κB activity and effectively slowing the inflammation (132). In addition, an edible plant (i.e., ginger)-derived exosome-like nanoparticle (ELN) RNA has been shown to induce IL-22 by activating the AHR pathway, thereby improving intestinal inflammation (133). Human umbilical cord-derived mesenchymal stem cell exosome-derived miRNA-181c regulates systemic inflammation by reducing TLR4 (toll-like receptor 4, LPS receptor) expression, causing decreased expression of p65/NF-κB phosphorylation, inhibiting TNF-α and IL-1β secretion, and increasing IL-10 secretion (10, 129) (**Figure 3**). Zhao et. also found that miR-140-5p in adipose tissue macrophage-derived exosomes can target SLC7A11 to inhibit GSH synthesis and then induce cardiac injury, which provides a clear and novel therapeutic strategy (134).

## 5 Conclusion and prospect

In inflammatory diseases, amino acid metabolism is disturbed by the disease state or inflammation, mainly manifested in the reduction of absorption and utilization of most amino acids, decrease of related intestinal flora, and tissue functional damage. Both neutral amino acids or aromatic amino acids or aromatic amino acid transport proteins are destroyed to varying degrees. In addition to tryptophan, glutamine, and arginine also play an important role in the anti-inflammatory process. Most studies have observed the improvement of these exogenous amino acids by adding one or multiple dietary amino acids, most of which are animal experiments. An improvement in inflammation can indeed be observed, but the specific mechanisms need to be thoroughly studied. The link between inflammation and amino acid metabolism goes through key nodes, such as MAPK, NF-κB, and other signaling pathways, as well as key immune cells.

Amino acid metabolism is involved in the various anti-inflammatory mechanisms of exosomes. Mesenchymal stem cells and their derived exosomes directly or indirectly regulate key signaling pathways in inflammation, such as NF-κB and STAT. This is only the anti-inflammatory mechanism of the large framework, under which the amino acid metabolism constantly operates and functions in the regulation and being regulated. Exosomes can even directly regulate the activity of the AHR, acting on intestinal inflammation by controlling the Trp-AHR pathway. Some components of exosomes, such as non-coding RNA, are significant in anti-inflammatory processes and are easily linked to amino acid metabolism by regulating pathways such as NF-κB, which is well worth further exploration in later studies.

Although some assumptions have not been directly demonstrated for the time being, there are other emerging treatments for IBD, such as fecal flora transplantation, dietary fiber intake, and other methods that have been effectively employed in the treatment of IBD, from the perspective of amino acid metabolism. The functions of many components of exosomes exhibit good feedback in the treatment and anti-inflammation of IBD, but there are still many mechanisms and pathways that are not fully understood. We hope that there will be more practical evidence for the involvement of amino acid metabolism in the treatment of IBD in future studies.

## Author contributions

XZ: Writing – original draft. YZ: Writing – original draft. ZZ: Writing – review & editing. YC: Funding acquisition, Writing – review & editing. WY: Conceptualization, Writing – original draft.
